# Supplementary material for: TIR1/AFB-Aux/IAA auxin perception mediates rapid cell wall acidification and growth of Arabidopsis hypocotyls
Source: eLife. 2016 Sep 14;5:e19048. doi: 10.7554/eLife.19048 (PMC5045290; doi:10.7554/eLife.19048)
Supplement: Supplementary file 1. — DOI: http://dx.doi.org/10.7554/eLife.19048.017 [file elife-19048-supp1.docx]

The AreKymo script, to be pasted into MATLAB as a script:

____________________________________________________________

%areaKymo

%inputs are 16 bit 2-channel, multitime tif images with green channel first, red

%channel second. Red channel is the reference channel - works for GFP/RFP

%pH sensors, but should also work for R2D2-type sensors.

%I use SUM projection of z-stacks - these are 32 bit, convert carefully to

%16bit, do not loose information during the conversion.

%orient the picture in the way that the interesting features are aligned

%vertically as much as possible

%find out the threshold when your red channels is well thresholded - you see just the feature you care about, in my case cell walls.

% input this as threshold.

clear all; close all;

%parameters

%________________________________________________________

threshold= 14000; %set manually based on the red channel. set threshold=-1 to automatically determine the threshold

twidth=15; %define the length of single timepoint in pixels = twidth

ratioRange=[0 2]; % y range in box plot

ratioLimit=2; % limit the maximum Ratio to this value, set ratioLimit=Inf if you don not want to limit the Ratio

% limiting the ratio is useful if the images are very noisy

%________________________________________________________

[sName,sNewPath]= uigetfile('*.tif','Select the image');

cd(sNewPath);

info = imfinfo(sName);

dimx=info(1).Width;

dimy=info(1).Height;

xres=info(1).XResolution;

yres=info(1).YResolution;

nt = numel(info)/2;

g=zeros(dimy,dimx, nt);

r=zeros(dimy,dimx, nt);

disp('reading image file ... stay tuned ');

for k = 1:nt

g(:,:,k)=imread(sName, 2*k-1, 'Info', info);

r(:,:,k)=imread(sName, 2*k, 'Info', info);

end

figure(1);

imshow(imfuse(r(:,:,1),g(:,:,1),'falsecolor','ColorChannels',[1 2 0]));

title(sName);

minE=Inf;

for k = 1:nt

im1=r(:,:,k);

m1=im1(im1>threshold);

if numel(m1)<minE

minE=numel(m1);

end;

end;

basewidth = fix(minE/twidth);

minE=twidth*basewidth;

subimsize=[basewidth minE/basewidth];

r1=zeros([ subimsize(1) subimsize(2)*nt]);

g1=r1;

if threshold<0

autothresh=multithresh(r(:,:,1),3);

threshold=autothresh(end);

end;

for k = 1:nt

im1=r(:,:,k);

im2=g(:,:,k);

m1=im1(im1>threshold);

m2=im2(im1>threshold);

r1(:,(k-1)*subimsize(2)+1:k*subimsize(2))=reshape(m1(1:minE),subimsize);

g1(:,(k-1)*subimsize(2)+1:k*subimsize(2))=reshape(m2(1:minE),subimsize);

end;

figure(2);

%calculate the ratio image, best visualized by the Fire LUT

ratio=r1./g1*10000; %mutliply with 10000 to save as 16bit tif

imwrite(uint16(ratio'),[sName(1:end-4) '_ratioRG.tif']);

imshow([sName(1:end-4) '_ratioRG.tif']);

title('Ratio red/green');

colormap hot;

figure(3); %this one shows the GFP and RFP channels, mind that the LUT of the RED must be changed, otherwise starts at the threshold.

maxint=max([max(max(r1)) max(max(g1))]);

r1=r1./maxint*65535;

g1=g1./maxint*65535;

imshow(imfuse(r1',g1','falsecolor','ColorChannels',[1 2 0]));

title('Overlay Red+Green');

imwrite(uint16(r1'),[sName(1:end-4) '_r.tif']);

imwrite(uint16(g1'),[sName(1:end-4) '_g.tif']);

disp('calculating ratios ... ');

%get the numbers from the total (non shrunk) thresholded images, calculate the ratio

%between Green to Red

TOT = [];

grp = [];

for k = 1:nt

Red1=r(:,:,k);

Gre2=g(:,:,k);

Red=Red1(Red1>threshold);

Gre=Gre2(Red1>threshold);

Rat=Gre./Red;

Rat(Rat>ratioLimit)=ratioLimit;

grp = [grp,k*ones(1,numel(Rat))];

TOT= [TOT;Rat];

end;

fig = figure;

h=boxplot(TOT,grp);

set(h(7,:),'Visible','off') %remove outliers

ylim(ratioRange); %the range can be specified...

title('Ratio Green/red (t)');

xlabel('frame #');

ylabel('Ratio');

print(fig,[sName(1:end-4) '_boxplot'],'-dpng');

disp('done ... ');
